# Challenges in the interdisciplinary treatment of leakages after left-sided colorectal surgery: endoscopic negative pressure therapy, open-pore film drainage therapy and beyond

**DOI:** 10.1007/s00384-023-04418-5

**Published:** 2023-05-19

**Authors:** Pasquale Scognamiglio, Anja Seeger, Matthias Reeh, Nathaniel Melling, Karl F Karstens, Thomas Rösch, Jakob R Izbicki, Marcus Kantowski, Michael Tachezy

**Affiliations:** 1 Department of General, Visceral and Thoracic Surgery, University-Hospital Hamburg-Eppendorf, Martini Str. 52, 20246 Hamburg, Germany; 2 Elisabethinum Medical Care Center, Hamburg, Germany; 3 Clinic of Interdisciplinary Endoscopy, University Hospital Hamburg-Eppendorf, Hamburg, Germany

**Keywords:** Leakage, Complication management, Vacuum therapy, Surgery, Rectal cancer

## Abstract

**Purpose:**

The treatment of anastomotic leakage after left colorectal surgery remains challenging. Since its introduction, endoscopic negative pressure therapy (ENPT) has proven to be advantageous, reducing the necessity of surgical revision. The aim of our study is to present our experience with endoscopic treatment of colorectal leakages and to identify potential factors influencing treatment outcome.

**Methods:**

Patients who underwent endoscopic treatment of colorectal leakage were retrospectively analyzed. Primary endpoint was the healing rate and success of endoscopic therapy.

**Results:**

We identified 59 patients treated with ENPT between January 2009 and December 2019. The overall closure rate was 83%, whereas only 60% of the patients were successfully treated with ENPT and 23% needed further surgery. The time between diagnosis of leakage and uptake of endoscopic treatment did not influence the closure rate, but patients with chronic fistula (> 4 weeks) showed a significantly higher reoperation rate than those with an acute fistula (94% vs 6%,* p* = 0.01).

**Conclusion:**

ENPT is a successful treatment option for colorectal leakages, which appears to be more favorable when started early. Further studies are still needed to better describe its healing potential, but it deserves an integral role in the interdisciplinary treatment of anastomotic leakages.

## Introduction

Anastomotic or stump leakage of left-sided colorectal resections is a frequent complication and its treatment remains challenging. After decades of surgical revision as primary approach, a paradigm shift has taken place to non-surgical interventional, mainly endoscopic regimes. Especially since the introduction of endoscopic negative pressure therapy (ENPT) in 2003 by Weidenhagen and colleagues, the majority of contained leakages are successfully manageable without surgical revision [1–5]. The published healing rates in large retrospective series are reported to lie around 90% (range 67–100%); with few therapy-related adverse events, endoscopic treatment is nowadays the gold standard in many colorectal centers [6–8]. Still, a significant number of patients cannot be successfully treated at first attempt using standard sponge therapy and develop pelvic abscesses or chronic leakages that significantly limit the patient’s quality of life and prevent ostomy closure.

Recently, several innovations and advancements in under pressure therapy have been introduced into the treatment of colorectal leakages allowing for adaption of the therapy to individual situations, localization, size, and healing status of the leak. This includes developments in negative pressure therapy, such as open-pore film drainage (OFD) and individualized sponges, but also in combination with established methods such as clipping, suturing, fistula plug insertion, closure with glue, and others [6, 9–12]. Recently, de Moura and colleagues developed a less expensive, but equally effective alternative [13]. In other reports, transanal rinsing therapy (TRT) was administered during and after endoscopic therapy [14].

The aim of the current study is to analyze and present our methods and experience in the treatment of patients with leakages after colorectal surgery focusing on second-line treatment of refractory cases and to identify potential factors influencing the success of the endoscopic therapies.

## Results

Between January 2009 and December 2019, 59 patients with postoperative leakage after colorectal resection were treated with ENPT in the Interdisciplinary Endoscopy Department of the University Medical Center Hamburg-Eppendorf. Patient demographics and clinic characteristics are listed in Table 1.

**Table 1:** Patient characteristics

		Median or total number	(%)
Total		59	(100)
Age (median and range)		59.6	(11/77)
Sex (*n*)	Male	38	(63)
	Female	21	(37)
Diagnosis (*n*)	Colorectal carcinoma	33	(57)
	Other carcinoma	6	(10)
	Diverticular disease	6	(10)
	Chronic inflammatory disease	4	(7)
	Other	10	(16)
Anastomosis (*n*)	Ileo/colorectal anastomosis	45	(77)
	Hartmann procedure	14	(23)
Neoadjuvant RCTx/CTx (n)	Yes	21	(37)
	No	35	(58)
Healing rate	Overall	49	(83)
	ENPT	35	(60)
	ENPT + Surgery	14	(23)
Mortality		3	(5)

*RCTx* radiochemotherapy, *CTx* chemotherapy

The primary surgery was performed for colorectal cancer in the majority of the cases (*n* = 33, 57%). Of the remaining patients, six (10%) suffered from diverticular disease, six (10%) underwent colorectal resection as part of an operation for other malignancies, four (7%) suffered from chronic inflammatory disease, and 10 (16%) presented with other pathologies. In regard to the reconstruction, most patients (*n* = 45, 77%) received colorectal or ileorectal anastomoses with protective ostomies, whereas the remaining patients (*n* = 14, 23%) underwent Hartmann’s procedure with a terminal ostomy. Twenty-one (37%) patients underwent neoadjuvant (chemo)radiation.

Three patients (5%) died within 30 days after start of ENPT: two as a consequence of septic complications and one due to systemic tumor progression.

OFD was used in 15 patients, either with (*n* = 8) or without (*n* = 7) an additional sponge. The sponge alone was the first-line treatment in 27 patients. OFD was used as additive treatment in three patients, second line treatment in 3 patients and 2 patients received an OFD treatment alone in the beginning of their therapy and then needed additive sponge treatment. Overall, 24 patients received additional TRT either by daily rectal enema, the use of special rinsing catheters [14], or regular irrigation systems (Peristeen, Coloplast, Germany).

The endoscopic treatment was started at a median of 18 days after surgery (range 2–3724) and took a median time of 18 days (range 3–86). A median of 4 endoscopies per patient were performed (range 1–13).

### Therapy-related complications

Complications occur in 14 out of 59 (20%) patients and are summarized in Table 2. Four patients developed a stenosis of the anastomosis, needing further endoscopic treatment. No short-term ENPT-related complications occurred. Six patients developed symptoms of low anterior resection syndrome (LARS). Other complications included one chronic pouchitis and one symphysitis. The different techniques and materials used did not show any differences in terms of complications.

**Table 2:** Treatment-related complications

		Total number	(%)
Total (*n*)		14	24
ENPT-related complications			
Stenosis of the anastomosis		4	7
Other complications			
Sepsis		1	2
Pneumonia		1	2
Chronic pouchitis		1	2
Symphysitis		1	2
LARS		6	10
	Sphincter dysfunction	4	7
	Diarrhea	1	2
	Obstipation	1	2

*ENPT* Endoscopic negative pressure therapy, *LARS* lower anterior resection syndrome

### Factors associated with successful treatment

Successful treatment was defined as complete closure of the leakage. This was reached in 49 patients (83%). Of those, 35 (60%) were successfully treated with ENPT, whereas 14 (23%) underwent redo surgery to achieve complete healing of the leakage.

Since no differences were detected between the endoscopic negative pressure devices (ENPT vs ENPT/TRT and OFD with or without sponge vs sponge alone), we analyzed which other factors might have influence on healing rates. Results are summarized in Table 3.

**Table 3:** Factors influencing treatment success

		**Successful healing**		**Treatment failure**		
		Median or total number	(%)	Median or total number	(%)	Significance (*p*)
Total		35	(100)	24	(100)	
Age (median and range)		60	(30/75)	60	(11/78)	nS
Sex (*n*)	Male	22	(63)	16	(67)	
	Female	13	(37)	8	(33)	nS
Diagnosis (*n*)	Colorectal carcinoma	21	(60)	12	(50)	
	Other carcinoma	4	(11)	4	(16)	
	Diverticular disease	4	(11)	2	(8)	
	IBD	1	(2)	3	(13)	
	Other	5	(16)	3	(13)	nS
Anastomosis (*n*)	Ileo/colorectal anastomosis	31	(87)	14	(58)	
	Hartmann procedure	4	(13)	10	(42)	**.041**
Leakage (*n*)	Anastomotic leakage	30	(83)	7	(29)	
	Rectal stump leakage	3	(11)	8	(33)	
	Pelvic abscess	1	(3)	4	(18)	
	Fistula with other organs	1	(3)	5	(20)	**.002**
Neoadjuvant RCTx/CTx (n)	Yes	11	(34)	12	(50)	
	No	24	(66)	12	(50)	nS
Reoperation	Yes	18	(51)	7	(29)	
	No	17	(49)	17	(71)	nS
Treatment duration (days)		18		26		nS
Time to endoscopic therapy	Acute leakage (< 4 weeks from diagnosis)	16	(46)	7	(29)	
	Chronic leakage (> 4 weeks from diagnosis)	19	(54)	17	(7)	nS
OFD	Yes	8	(23)	7	(29)	
	No	27	(77)	17	(71)	nS
Sponge	Yes	31	(89)	21	(88)	
	No	4	(11)	3	(12)	nS
TRT	Yes	16	(46)	8	(33)	
	No	19	(54)	16	(64)	nS

Due to the retrospective character of the study, not all numbers sum up to the total number of patients

*IBD* inflammatory bowel disease, *RCTx* radiochemotherapy, *CTx* chemotherapy, *TRT* transanal rinsing therapy, *OFD* open-pore film drainage

Patients with ileorectal or colorectal anastomosis had a better healing rate than the ones with terminal ostomy (*p* = 0.041). The kind of leakage also played a role on treatment success, with an isolated anastomotic leakage being more likely to heal than pelvic abscess or fistula to other organs (*p* = 0.002).

However, neither the size of the abscess nor other factors like the indication for surgery, neoadjuvant radiation, type of operation, reoperation during endoscopic treatment, treatment duration and time between leakage diagnosis and start of ENPT revealed at correlation to treatment success.

**Fig. 1:**
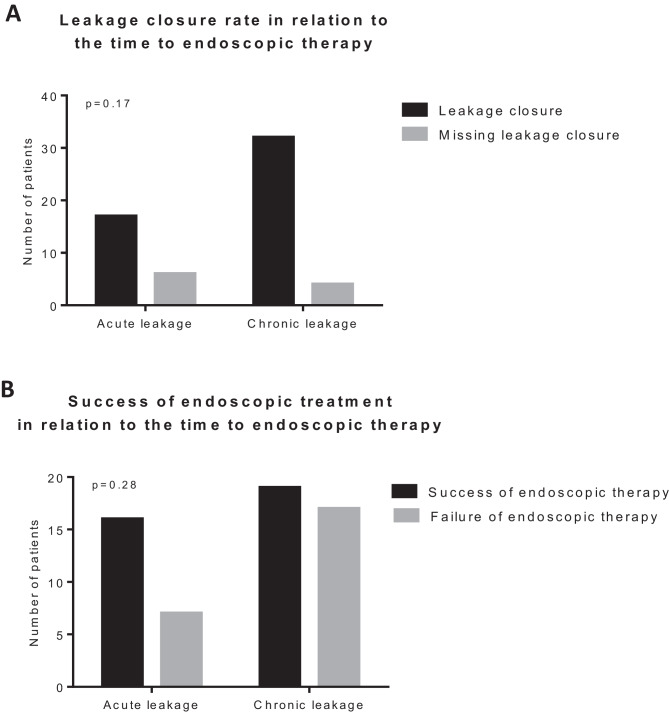
**A** Leakage closure rate in relation to the time to endoscopic therapy. *p* value was determined with the Fisher exact test. **B** Success of endoscopic treatment in relation to the time to endoscopy. *p* value was determined with the Fisher exact test

**Fig. 2:**
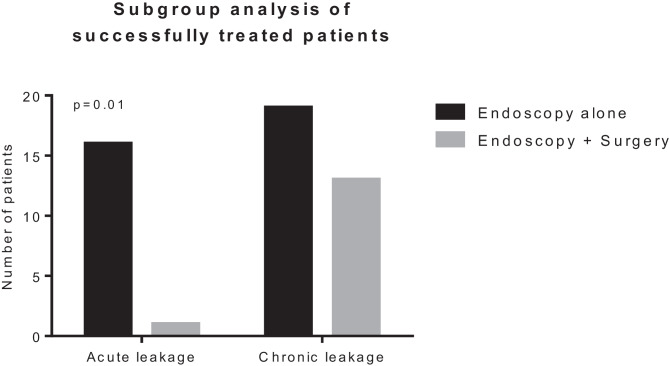
Subgroup analysis of successfully treated patients: comparison of re-operation rate in relation to the time to endoscopic treatment. *p* value was determined with the Fisher exact test

Since more than the half of the patients in our cohort (*n* = 32, 54%) presented to endoscopic therapy with a chronic leak (longer than 4 weeks after diagnosis), we questioned whether the time between diagnosis of leakage and beginning of endoscopic therapy influenced the outcome of the therapy. We divided the patients into two groups based on the time elapsed till endoscopic therapy started: acute leakage (< 4 weeks) and chronic leakage (> 4 weeks). The time to endoscopic therapy failed to show an effect on both overall closure rate (Fig. 1a) and success of endoscopic therapy (Fig. 1b). Furthermore, we performed a subgroup analysis of the successfully treated patients. Interestingly, only 1 out of 17 patients with an acute leakage (6%) needed redo surgery to achieve complete healing. The reoperation rate was significantly higher (13/32, 41%, *p* = 0.01) for chronic leakages (Fig. 2).

## Discussion

Even though ENPT is well established for treatment of anastomotic leakages after colorectal surgery with a success rate of 81%, a subset of patients develops complications after termination of the therapy, such as recurrent abscess or fistula [7]. Thus, several modifications of standard ENPT have been described in literature and the necessity for individualized treatment becomes more evident [11, 13].

**Fig. 3:**
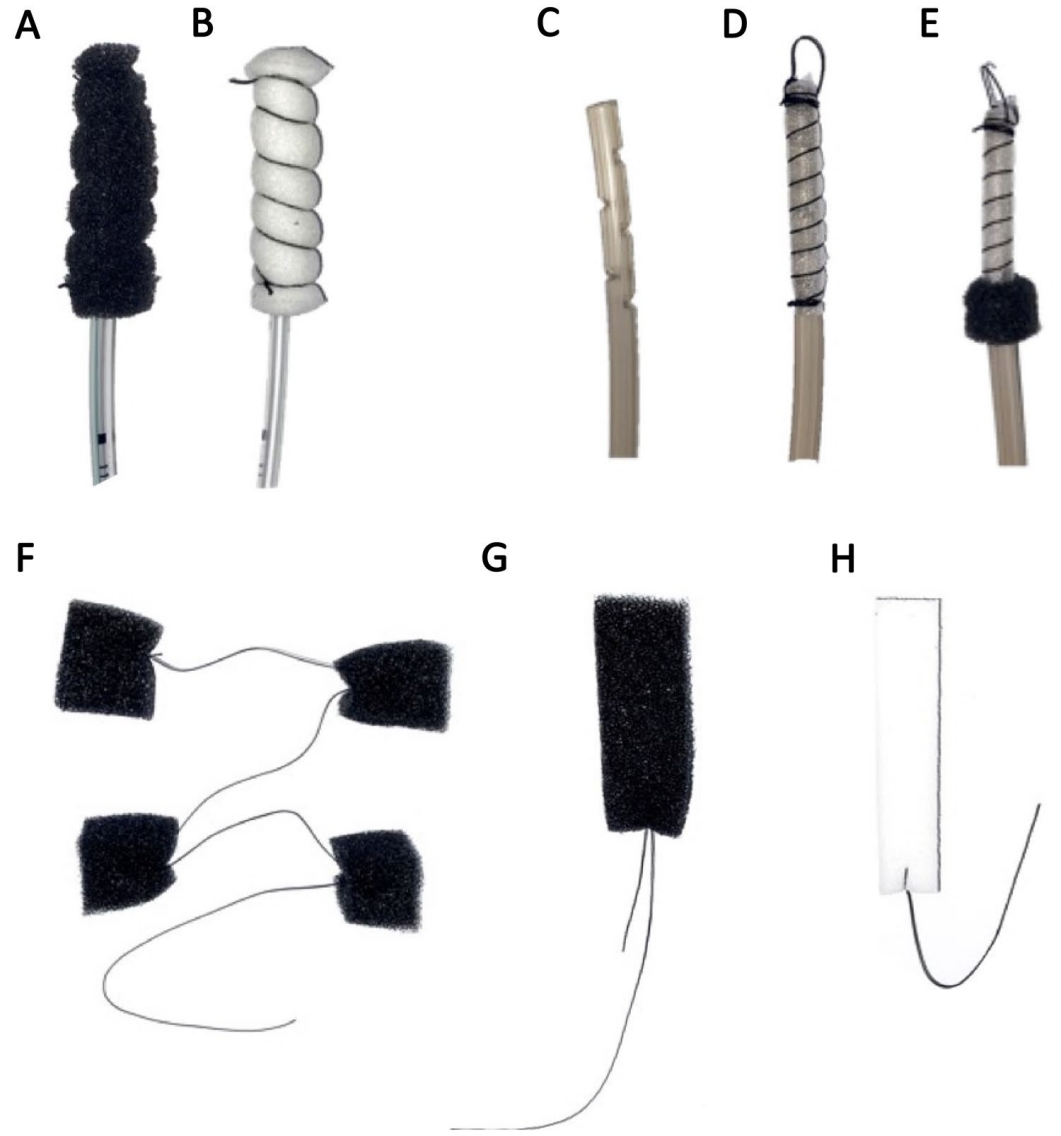
Materials used for ENPT in our cohort: **A** Black sponge; **B** White sponge; **C** Gastric tube with side holes, **D** covered with a double-layered, multiperforated, open-pore film (**E**) with and additional black sponge; **F** string of pearls (individually cu sponge cubes connected with a non-absorbable, braided suture; **G** black sponge and **H** White sponge fixed with a suture, allowing an easier removal

The different materials available offer the possibility to customize the treatment to each individual’s anatomic situation. For example, for long, narrow abscess formations, sponges may be too voluminous. Here, the OFD presents an appropriate alternative [12]. In our study, we successfully used OFD both for first-line and for additive treatment. In larger abscesses, we use more foam material, as shown in Fig. 3F to induce granulation all over the cavity. Depending on the state of the cavity, either black or white sponges can be inserted. The latter can be left in place for a longer period due to its reduced ingrowth capacity. Therefore, we often use white sponge material or OFD for outpatient treatment to have more flexibility regarding the changing interval. Another recent development is the combination of OFD and black sponge in which the black sponges serves as a “shield” towards the bowel (Fig. 3E). The black sponge covers the orifice, adheres strongly due to its properties, and impedes fecal contamination of the fistula or abscess behind the bowel wall. Usually, the abscess cavity collapses under the negative pressure, and a long and narrow canal is formed which can be reduced step by step until closure is achieved.

In this study, we are able to report on an overall closure rate of 83%. However, only 60% reached complete closure of the leakage with ENPT therapy alone, whereas the remaining 23% underwent further surgery. Our treatment result is at the lower end of the success rates (56–97%) published in the literature so far [7]. We therefore critically re-evaluated our cohort of patients in order to find out which factors might influence the outcome of patients with colorectal anastomotic leakage.

Patients receiving an ileorectal or colorectal anastomosis with protective ostomy fashioned during the primary operation showed higher healing rates than those with a terminal ostomy. This may be due to the fact that the decision-making in favor of terminal ostomy is mostly influenced by peritonitis or relevant co-morbidities such as immuno-suppression, which per se represent a risk factor for leakage [15]. Accordingly, simple anastomotic leakage also proved to heal better compared to pelvic abscesses or fistulas to other organs.

Another important aspect for the ENPT is the localization of the defect: the treatment of intrapelvic abscesses is safer, since the negative pressure is not directly applied on the abdominal cavity, but in our experience, contained abscess cavity can be treated with ENPT even in extrapelvic cases.

Moreover, more than the half of our patients presented with a chronic fistula, some of them persisting for many years. Although falling short of significance, the closure rate after ENPT was higher in the group with early treatment start compared to the chronic fistula group (70% versus 53%, *p* = 0.28). This is in line with the large cohort described by Kühn and colleagues, who were also not able to find a difference between the early and late onset of the therapy [8]. In the small series published by Van Koperen and colleagues, a significantly lower healing rate was seen after a long interval between the index operation and the start of the EPNT [16]. In the sub-analysis of the successfully treated patients, the re-operation rate was significantly higher in the chronic compared to the acute leakage group (41% vs 6%, *p* = 0.01). On the one hand, these results underline the importance of a prompt beginning of the endoscopic therapy to avoid re-operations. On the other hand, it suggests that ENPT has a healing potential of approximately 50% for chronic leakages. Therefore, it might well be worth a try before going back for surgery considering revision surgery’s high potential for morbidity. Moreover, our results underline the importance of an early leakage diagnosis. In this regard, routinely early postoperative endoscopic evaluation of rectal anastomosis was recently shown to significantly anticipate leakage diagnosis and improve the clinical course and should be considered as standard after colorectal surgery [17].

Rinsing endoscopic therapy without ENPT is also gaining interest: as an example, Shalby et al. described a technique of a balloon-blocked transanal drainage without ENPT, showing good results [9]. In our cohort of patients, we used TRT as an additive treatment during ENPT for colorectal leakages (ENPT/TRT) as described in our previous work [14]. This can be used by the patients on their own on an out-patient basis, which is of importance since outpatient treatment has been shown to be associated with a higher success rate [8].

In their recent retrospective study on 281 patients treated with ENPT for colorectal leakage, Kühn et al. reported the following factors as significantly negatively influencing the success of ENPT: [1] multivisceral resections, [2] recent surgical revision, and [3] treatment duration. In our study, no patient had undergone multivisceral resections. Surgical revisions and treatment duration show a tendency towards unfavorable results, but do not reach statistical significance, probably due to the smaller amount of patients included in our analysis.

Usually, treatment duration and repeated endoscopies with associated sedations and periprocedural stress for the patients are one of the major concerns regarding ENPT. Median treatment duration in our cohort was 18 days, which is shorter than the median 31 and 47 days that were recently reported in meta-analyses [7, 18]. Correspondingly, we also report on a median of 4 endoscopic procedures per patient which is less than the published average of 7 procedures [7, 18]. In our sub-analysis, patients with chronic fistula proved to need a longer treatment and a higher number of interventions than those with acute fistula (respectively, 23, 5 vs 15 days, and 5 vs 3 interventions), but the difference was not statistically significant (data not shown). Nevertheless, repeated interventions allow endoscopic lavages and debridement at every session, thus reducing perianastomotic inflammation [7]. A relevant number of patients can be discharged from hospital and treated on an outpatient basis [8]. So, in summary, the length of treatment and low complication rate seem to overweigh the risk of the less successful conservative and much more harmful surgical treatment variants [19].

Fourteen patients developed complications in our cohort. The most frequent complications were stenosis of the anastomosis, which is in accordance with published literature [7]. However, stenosis is mostly not a consequence of the treatment, but of relative ischemia in the anastomosis region [20]. Two patients died of septic complications, not ENPT-related, whereas one patient died as a consequence of tumor progression. A relevant number of patients suffered from LARS that has a known increased risk after anastomotic leakage [21, 22]. Beside anastomotic stenosis, potential treatment-related complications might be bleeding complications or remaining fistula or abscesses/sinus; however, these were not present in our cohort [7]. Nevertheless, based on our experience, bleeding risk based on sponge ingrowth can be reduced with shorter changing intervals and by using fine pored or silicone coated sponges [23–25].

The retrospective design of our study and the size of the cohort does not allow us to give clear recommendations for the treatment of leakages after colorectal surgery. The extension of the ENPT armamentarium helps to individualize therapy for each patient, and each leak might also further optimize the results of this dangerous complication. ENPT is a safe and valid option for the treatment of patients with colorectal leakage.

## Materials and methods

### Patients and clinical data

A total of 59 patients with a radiologically and/or endoscopically confirmed colorectal leak with pelvic abscess (grade B according to the International Study Group of Rectal Cancer) were identified between 2009 and 2019 in the University Medical Center Hamburg-Eppendorf, Germany, prospective colorectal database. Patients needing reoperation due to colonic ischemia proximal to the anastomosis or diffuse peritonitis were excluded from our analysis.

The study was approved by the Ethics Committee of the Medical Chamber Hamburg and was performed in accordance with the ethical standards laid down in the 1964 Helsinki Declaration and its later amendments. Informed consent was obtained from all patients before study inclusion.

All clinical data were collected from a combination of clinical and endoscopic record reviews and communication with patients and their attending physicians. The data obtained included leakage closure, recurrence of pelvic abscess and sepsis, rate of ostomy closure, and therapy-related complications.

### Leakage diagnosis

**Fig. 4:**
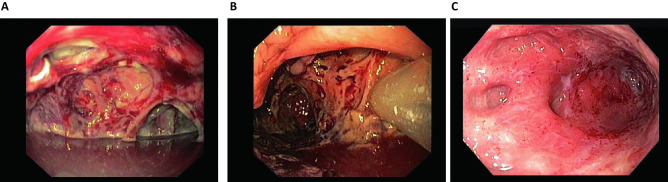
Endoscopic finding of leakage at diagnosis (**A**), during ENPT (**B**) and after leakage closure (**C**)

In cases of postoperatively increased inflammation markers, fever, fascial dehiscence, perianal pus, and fecal or purulent discharge in drains or signs of deterioration, leakage was suspected. Endoscopic examinations and (in case of clinical signs of a larger cavity) CT scans were performed to confirm the leak and to verify presence of an abscess or generalized peritonitis. If leakage or abscess were confirmed and found to be contained in the pelvic region, ENPT was initiated. During the first endoscopic treatment session, the large bowel was rinsed and mechanically cleaned and information on size of the leakage, and the abscess, bowel vitality, visible vessels, or bowel contained in the abscess cavity was collected (Fig. 4A). Usually a standard gastroscope was used, but in cases of very small leakages, a 5.6-mm fine caliber transnasal gastroscope (GIF XP 290N, Olympus) was used. An endoscopic exploration through the defect was only performed when the defect was big enough and the small endoscope was available. In case of a large and dirty abscess, we dilated the orifice with a balloon or with a finger to insert a sponge extraluminally in order to reach a significant better cleaning, granulation and closure of the leakage.

### Endoscopic negative pressure therapy (ENPT)

Depending on the size of the leak, the size of the abscess, and the grade of contamination, different materials for the application of under-pressure are available. While the black sponge (3 M™ V.A.C.^®^ Granufoam ™,3 M St. Paul, MIN) with its relatively large pores proves good wound cleansing properties and granulation stimulus, early ingrowth can be observed and therefore it must be removed after 2–5 days. This is in contrast to the white sponge material (3 M™ V.A.C.^®^ Granufoam™, 3 M St. Paul, MIN) which can be used in critical proximity to bigger vessels, thus avoiding bleeding complications, or in case of free contact of the sponge bowel, so as to reduce the risk of intestinal erosion (e.g., by ENPT in the abdominal cavity). OFD (Suprasorb CNP by Lohmann & Rauscher, Germany) was applied in small diameter fistula-like defects where there was not enough room to place a sponge. These open-pore film catheters can be left in place for a longer time interval. In our setting, we change them once a week.

1. ***“Classical” endo sponge***
  After endoscopic exploration of the leakage and the pelvic cavity, an extraluminal, intracavitary sponge is inserted into the abscess (Endo- or Eso- Sponge^®^, B. Braun Melsungen, Germany). The placement of the sponge is usually performed via an overtube, but in cases with difficult access to the abscess cavity, the drawstring method is used [11]. In small leakages with relatively clean abscesses, the sponge can be placed intraluminally at the height of the orifice to initiate collapsing of the abscess.
2. ***Individually fitted sponge drainages and “string of pearls”***
  When treating smaller leakages or small openings into the paraluminal abscess, either the amount of sponge material was reduced by cutting to size or customized sponges (as already described) with either black or white sponge material were used (3 M St. Paul, MIN) (Fig. 3A, B) [26, 27].
  In cases with very large abscesses, we introduced additional customized sponge cubes into the cavity that were either connected to each other with a non-absorbable, braided suture (“string of pearls”) or a suture was fixed to each sponge segment so as to facilitate the removal (Fig. 3F–H).
3. ***Open-pore film drainage***
  OFD was manufactured as recently described [12, 28]. Briefly, a gastric tube between 12 and 18 Charriere (Ch) or shortened TRAC pad adapter for the KCI pump (3 M St. Paul, MIN) is required for the construction. Size is adapted to the size of the leakage or the remaining channel. According to the findings, side holes are cut into the tube over a length of about 5 cm starting at the distal tip of the probe (Fig. 3C). This distal segment is covered with a thin, double-layered, multiperforated, open-pore film (Suprasorb CNP by Lohmann & Rauscher, Germany) and fixed with sutures (Fig. 3D). In some cases, another black sponge segment was added at the proximal end of the open-pore film cover and positioned at the leakage’s orifice (Fig. 3E). This probe with an open-pore film on the tip can be placed with a distal trailing thread, wire, or forceps.

### Negative pressure application methods and changing of the suction material

Negative pressure was obtained by an electronic pump (3 M, St. Paul, MIN) with a pressure of − 125 mmHg, or (when not available) a Redyrob bottle from the B. Braun standard kit using level one (− 100 mmHg). Endoscopic changing of the sponge was performed twice a week, in cases with white sponges or OFDs, after up to 8 days. Depending on the grade of necrotic tissue, either rinsing of the cavity and/or mechanical debridement with a forceps or brush was performed.

The treatment was terminated once stable clinical conditions and a clean abscess cavity with granulation tissue were achieved (Fig. 4B). In patients with a remaining wound cavity at the site of the leak, daily TRT was administered, as described in our previous work [14]. A period of 3 months between endoscopically confirmed healing of the abscess cavity after ENPT, and ostomy closure to avoid recurrent abscess was aimed at. A few days before ostomy closure, a control endoscopy was performed with assessment of the healing status (Fig. 4C).

### Treatment failure

Failure of endoscopic treatment was defined as lack of visible healing or granulation process after 4 to 6 weeks of treatment.

### Statistical analysis

All statistical analyses were carried out using IBM^©^ SPSS^©^ Statistics for Mac (Version 20, IBM Corporation, Armonk, NY, USA). The median with interquartile distance and the mean value with minimum and maximum were used to describe the patients. After exploration, statistical evaluation of continuous data was performed using the non-parametric Mann–Whitney test. For categorical data, the χ^2^ test and Fisher exact test were used. All tests were two-sided, and statistical significance was set to *p* < 0.05.

## Data Availability

The datasets generated during and/or analyzed during the current study are available from the corresponding author on reasonable request.
